# Assessing Radiation Dosage in Pediatric Head and Neck Computed Tomography Examinations During COVID-19 in a Tertiary Hospital in Saudi Arabia, Jeddah

**DOI:** 10.7759/cureus.33588

**Published:** 2023-01-10

**Authors:** Mawya Khafaji, Abdulrahman Y AlNajjar, Sultan Matbouli, Yasser A Alnahdi, Lama H Meriky, Sara Hagi

**Affiliations:** 1 Radiology, King Abdulaziz University Faculty of Medicine, Jeddah, SAU; 2 Medical School, King Abdulaziz University Faculty of Medicine, Jeddah, SAU; 3 Radiology, King Abdulaziz University Hospital, Jeddah, SAU

**Keywords:** radiation, exposure, computed tomography, pediatric, radiation dose

## Abstract

This study aimed to assess the practice of imaging and optimization of the radiation dose in pediatric head and neck computed tomography (CT) examinations during the coronavirus disease of 2019 (COVID-19) period. This study is based on a retrospective analysis of pediatric head CT records, conducted in the Radiology Department of the King Abdulaziz University Hospital in Jeddah, Saudi Arabia. We examined the data of all pediatric patients between 0 and 14 years of age who underwent head CT scans between March and September in both 2019 (before the COVID-19 pandemic) and 2020 (during the COVID-19 pandemic). In total, we analyzed 1005 scans; 531 (52.8%) were performed before and 474 (47.2%) during COVID-19. The dose parameters were similar; however, the exposure time was significantly lower during COVID-19 (5432 ms vs. 5811 before; p < 0.001). In contrast, the mean total CTDI_vol_ and dose-length product (DLP) were slightly higher during COVID-19 than those before (23.34 mGy vs. 22.04 mGy (p-value=0.565) and 577.36 mGy*cm vs. 518.93 mGy*cm (p-value=0.193) respectively). These changes could be attributed to the desire to limit the contact between technicians and patients. The limitation of contact with the patient allows the technicians to be independent during the scan, possibly accounting for this slight decrease.

## Introduction

Computed tomography (CT) is a commonly used imaging modality for investigation and diagnosis in specific clinical situations [1]. Compared to other modalities exposing patients to ionizing radiations, such as radiography, CT is characterized by a high radiation dose. Therefore, it is considered worrisome for its high radiation-dose effects [2]. It has been reported that CT imaging accounts for approximately 11% of all procedures involving radiation exposure; furthermore, 70% of the medical-related radiation doses are caused by CT imaging [3]. "As low as reasonably achievable", i.e. (ALARA) is a principle that optimizes patient protection from unwanted radiation exposure [4]. Another important concept is that pediatric patients have a higher risk of experiencing radiation-induced lesions than adult patients. This risk is owing to children having rapidly increasing, dividing, and growing cells and organs, thus being more prone to cellular damage or mutations when radiation affects these cells [5]. A CT dose survey and a detailed analysis of pediatric patients in different age groups should be conducted for a deeper understanding and evaluation of the effects of local protocol parameters and patient size on the radiation doses [6].

In 2018, a retrospective study was conducted in the USA to examine single-phase pediatric non-contrast head CT scans (56% male patients and 53% children older than 10 years of age). The median CT dose index per volume (CTDI_vol_) was 33 mGy (interquartile range: 22-47 mGy) [7]. Similarly, a cross-sectional study in Japan in 2016 reported that the median head CTDI_vol _across 339 facilities, for the age groups of < 1, 1-5, and 6-10 years of age were 30.7 mGy, 36.1 mGy, and 47.8 mGy, respectively [8]. There was also a retrospective cohort study conducted in the Netherlands on 168,394 children, which found that the mean cumulative brain dose was 38.5 mGy and that it was significantly associated with the risk of malignant and benign brain tumors [9]. Furthermore, a retrospective study in Malaysia evaluated 250 pediatric patients and found that the third quartile values among 134 pediatric patients were higher than the lowest values established as the diagnostic reference levels for head and neck CT scans [10].

In late 2019, severe acute respiratory syndrome coronavirus 2 (SARS-CoV-2), also known as coronavirus disease of 2019 (COVID-19), supposedly transmitted by bats, was first identified in humans in Wuhan, China [11]. As soon as the first few cases of COVID-19 were diagnosed and announced in Saudi Arabia on March 2, 2020, preventive measures were implemented by the Saudi Arabia Health Organization [12]. These measures were in accordance with the World Health Organization (WHO) recommendations, including mask-wearing, social distancing, and school lockdowns. Only emergency operations were performed in the medical settings; all clinics were limited, and virtual clinics were adopted in both public and private institutions. Although these restrictions were partially removed in June 2020, some were still implemented [13]. However, full curbs were lifted in September 2021, and this change was the basis of our data collection.

COVID-19 spread rapidly worldwide, resulting in more than 180,000 deaths and more than 2,600,000 infections by April 24, 2020 [14]. However, only 20% of all COVID-19-positive patients experienced severe symptoms [15]. COVID-19 has a mean incubation period estimated to be approximately five days post-exposure; however, some patients may remain asymptomatic for up to 19 days. Furthermore, all patients may transmit the virus during the incubation periods [12].

This study aimed to assess the practice of imaging and optimization of the radiation dose in pediatric head and neck computed tomography (CT) examinations during the COVID-19 period at the Radiology Department of the King Abdulaziz University Hospital in Jeddah, Saudi Arabia.

## Materials and methods

Study design

This study is a retrospective analysis of pediatric head computed tomography (CT) records, conducted in September 2021 in the Radiology Department of the King Abdulaziz University Hospital, and was approved by the corresponding Research Ethical Committee (reference No 132-22).

Subjects

A total of 1005 head CT scans were examined; the sample size was calculated using a confidence level of 95% and a margin of error of 6%. The study included all pediatric patients between 0 and 14 years of age who underwent head CT scans during the periods between March and September in both 2019 and 2020. 2019 scans served as the control group, whereas 2020 was the randomized comparison group. We analyzed the data from the CT scan protocols that were used in both periods (before and during COVID-19). The data were extracted in the form of a Microsoft Excel sheet from the radiation-dose-tracking software DoseWatch (version 2.0.4; GE Healthcare, Boston, MA, USA). This software collects and analyzes radiation and contrast dosage data across facilities and modalities to enable compliance.

Statistical analysis

The data were analyzed using SPSS version 24.0 (IBM, Armonk, NY, USA). Continuous data are reported as mean and SE or median and quartiles, as appropriate. Since the variables were not normally distributed, the non-parametric Mann-Whitney test was used to compare them before and during COVID-19. P-values < 0.05 (two-sided tests) were considered statistically significant.

Informed consent

The need for informed consent was waived due to the retrospective nature of the study.

## Results

Among the 1005 scans examined, 531 (52.8%) were performed before COVID-19, and 474 (47.2%) during the pandemic, with a 5.6% decrease.

Significant changes emerged in the exposure time, scanning length, and effective tube current-time product (effective mAs) before and during COVID-19. The exposure time, defined as the period of time when a volume is exposed to X-rays, showed a significant decrease during COVID-19 (5432 ms) compared to that in the period before (5811 ms; p < 0.001). The scanning length, that is the length between the first and last sections included in the scan was longer during COVID-19 than that before (215 mm vs. 208 mm; p = 0.008). Moreover, a significant increase was noted in the mean effective mAs during COVID-19 (237) compared to that before COVID-19 (164; p < 0.001). In contrast, the mean total dose-length product (DLP) and the CTDI_vol_ were slightly higher during COVID-19 than those before (577.36 mGy*cm vs. 518.93 mGy*cm and 23.34 mGy vs. 22.04 mGy, respectively; Table 1).

**Table 1:** Differences in the average radiation-dose parameters for all performed scans before and during COVID-19. n, sample number; SE, standard error; Total DLP, total dose-length product; CTDIvol, computed tomography dose index per volume The bold text represents significant p-values for the differences between before and during the COVID-19 pandemic.

	Before COVID-19 (n = 531)			During COVID-19 (n = 474)		P-value
	Mean	SE		Mean	SE	
Total DLP (mGy*cm)	518.93	15.41		577.36	26.99	0.193
Exposure time (ms)	5811	105		5432	138	< 0.001
Scanning length (mm)	208	4		215	3	0.008
Mean CTDI_vol_ (mGy)	22.04	0.52		23.34	1.27	0.565

The protocol used more frequently (n = 272 during COVID-19 and n = 207 before) was Head^HeadRoutine (Child), a CT scan without contrast used to diagnose or exclude hemorrhage, stroke, trauma, and mass occupying lesions; it is also used after shunt tube insertion. This protocol showed significant differences in total DLP (685.5 mGy*cm during COVID-19 vs. 645.6 before; p < 0.001), exposure time (5082 ms during COVID-19 vs. 5785 before; p < 0.001); scanning length (214 mm vs. 212 before; p = 0.01); CTDI_vol_ (26.78 mGy vs. 27.74 before; p = 0.014); and effective mAs (251 vs. 200 before; p < 0.001); the data are in Tables 2-6. In addition, Head^01_Head_helical protocol showed only significant differences in mean CTDI_vol_ (29.42 mGy vs. 41.04 before; p = 0.022) and effective mAs (113 vs. 166 before p = 0.022), which are demonstrated in Tables 5-6 respectively.

**Table 2:** Differences in total DLP before and during COVID-19 among several protocols. n, sample number; SE, standard error; total DLP, total dose-length product The bold text represents significant p-values for the differences between before and during the COVID-19 pandemic.

Imaging Protocol	Total DLP (mGy*cm) before COVID-19			Total DLP (mGy*cm) during COVID-19			P-value
	n	Mean	SE	n	Mean	SE	
BRAIN C-	38	775.4	33.5	20	1151.9	174.6	0.272
BRAIN C-/+	21	1181.2	72.6	13	1086.4	67.8	0.097
FACIAL BONES	1	869.9	.	2	963.2	0	0.157
Head^01_Head_helical	20	816.8	79	2	483.1	25.8	0.052
Head^01HeadRoutine (Ch)	48	549.3	19.3	20	549	38.2	0.904
Head^AAPM_HeadR_Spi_AEC	2	272.5	20.1	1	321.4	.	0.221
Head^Brain_SAFIRE (Ch)	5	324.4	49.1	20	340.4	30.4	0.838
Head^HeadRoutine (Ch)	207	645.6	12.8	272	685.5	35.4	< 0.001
Head^InnerEar (Ch)	40	162.9	6	10	192.1	41.7	0.286
Head^KAUH_Head_Fast_Routine	2	415.7	19.3	6	429.4	10.3	0.505
Head^NeonateHead (Ch)	5	462.7	96.8	6	359.1	33	1
NECK SOFT C-/+	13	468.4	90.9	15	389.2	66.7	0.533
Neck^KAUH_Neck_SAFIR	2	218.7	0	1	297.9	.	0.157
Neck^NeckRoutine (Adult)	1	356.9	.	2	259.3	56.2	0.221
Neck^NeckRoutine (Child)	16	103.5	19.7	6	153.9	31.9	0.077
Thorax^1_Chest_Routine (Ch)	3	224.9	0	1	94.5	.	0.083
Thorax^Flash_Thorax_SAFIR	2	52.3	6	5	96.1	11.8	0.087

**Table 3:** Differences in exposure time before and during COVID-19 among several protocols. n, sample number; SE, standard error; ms, milliseconds The bold text represents significant p-values for the differences between before and during the COVID-19 pandemic.

Imaging Protocol	Exposure time (ms) before COVID-19			Exposure time (ms) during COVID-19			P-value
	n	Mean	SE	n	Mean	SE	
BRAIN C-	38	5720	405	20	6626	521	0.437
BRAIN C-/+	21	4722	554	13	4150	608	0.71
FACIAL BONES	1	8160	.	2	6060	1250	0.221
Head^01_Head_helical	20	9035	203	2	8720	380	0.567
Head^01HeadRoutine (Ch)	48	6440	122	20	6370	192	0.423
Head^AAPM_HeadR_Spi_AEC	2	6615	685	1	7090	.	> 0.999
Head^Brain_SAFIRE (Ch)	5	6552	387	20	6349	189	0.838
Head^HeadRoutine (Ch)	207	5785	111	272	5082	215	< 0.001
Head^InnerEar (Ch)	40	2968	239	10	3095	603	0.619
Head^KAUH_Head_Fast_Routine	2	9125	95	6	9072	159	0.505
Head^NeonateHead (Ch)	5	5996	500	6	6007	260	> 0.999
NECK SOFT C-/+	13	4251	1085	15	5493	639	0.16
Neck^KAUH_Neck_SAFIR	2	6325	5325	1	10130	.	> 0.999
Neck^NeckRoutine (Child)	17	9459	718	8	10274	1129	0.658
Thorax^1_Chest_Routine (C	3	2480	990	1	2280	.	0.637
Thorax^Flash_Thorax_SAFIR	2	765	75	5	1256	688	0.324

**Table 4:** Differences in scanning length before and during COVID-19 among several protocols. n, sample number; SE, standard error; mm, millimeters The bold text represents significant p-values for the differences between before and during the COVID-19 pandemic.

Imaging Protocol	Scanning length (mm) before COVID-19			Scanning length during COVID-19			P-value
	n	Mean	SE	n	Mean	SE	
BRAIN C-	38	230	14	20	264	20	0.381
BRAIN C-/+	21	283	31	13	231	21	0.146
FACIAL BONES	1	297	.	2	251	26	0.221
Head^01_Head_helical	20	198	7	2	184	8	0.423
Head^01HeadRoutine (Child)	48	205	3	20	199	5	0.166
Head^AAPM_HeadR_Spi_AEC	2	220	4	1	218	.	> 0.999
Head^Brain_SAFIRE (Child)	5	201	12	20	201	5	0.812
Head^HeadRoutine (Child)	207	212	2	272	214	2	0.01
Head^InnerEar (Child)	40	82	9	10	85	23	0.619
Head^KAUH_Head_Fast_Routine	2	193	2	6	192	3	0.505
Head^NeonateHead (Child)	5	184	15	6	185	8	> 0.999
NECK SOFT C-/+	13	355	26	15	346	33	0.73
Neck^KAUH_Neck_SAFIR	2	182	177	1	350	.	> 0.999
Neck^NeckRoutine (Adult)	1	355	.	2	304	1	0.221
Neck^NeckRoutine (Child)	16	241	22	6	224	28	0.507
Thorax^1_Chest_Routine (Child)	3	250	123	1	245	.	0.637
Thorax^Flash_Thorax_SAFIR	2	311	30	5	166	64	0.049

**Table 5:** Differences in CDTIvol before and during COVID-19 among several protocols. n, sample number; SE, standard error; CTDIvol, computed tomography dose index per volume The bold text represents significant p-values for the differences between before and during the COVID-19 pandemic.

Imaging Protocol	Mean CTDIvol (mGy) before COVID-19			Mean CTDIvol (mGy) during COVID-19			P-value
	n	Mean	SE	n	Mean	SE	
BRAIN C-	38	15.54	2.21	20	19.29	3.05	0.277
BRAIN C-/+	21	16.75	2.94	13	16.49	3.16	0.79
FACIAL BONES	1	32.75	.	2	20.47	11.82	0.221
Head^01_Head_helical	20	41.04	1.96	2	29.42	0.13	0.022
Head^01HeadRoutine (Child)	48	30.51	0.71	20	29.02	1.23	0.258
Head^AAPM_HeadR_Spi_AEC	2	14.08	0.65	1	16.77	.	0.221
Head^Brain_SAFIRE (Child)	5	18.24	1.86	20	17.39	0.69	0.973
Head^HeadRoutine (Child)	207	27.47	0.37	272	26.78	1.86	0.014
Head^InnerEar (Child)	40	15.03	0.33	10	13.06	0.8	0.032
Head^KAUH_Head_Fast_Routine	2	23.88	0.87	6	24.87	0.58	0.402
Head^NeonateHead (Child)	5	28.25	3.23	6	22.17	1.26	0.068
NECK SOFT C-/+	13	5.35	1.34	15	5.93	1.75	0.765
Neck^KAUH_Neck_SAFIR	2	8.9	2.69	1	9.11	.	> 0.999
Neck^NeckRoutine (Child)	17	7.27	0.54	8	7.32	0.65	> 0.999
Thorax^1_Chest_Routine (Child)	3	3.11	0.12	1	4.31	.	0.157
Thorax^Flash_Thorax_SAFIR	2	1.67	0.04	5	3.32	1.27	0.051

**Table 6:** Differences in effective mAs before and during COVID-19 among several protocols. n, sample number; SE, standard error The bold text represents significant p-values for the differences between before and during the COVID-19 pandemic.

Imaging Protocol	Effective mAs before COVID-19			Effective mAs during COVID-19			P-value
	n	Mean	SE	n	Mean	SE	
BRAIN C-	38	131	10	20	152	17	0.216
BRAIN C-/+	21	203	31	13	266	40	0.261
FACIAL BONES	1	216	.	2	356	147	> 0.999
Head^01_Head_helical	20	166	12	2	113	1	0.022
Head^01HeadRout (Child)	48	179	5	20	168	6	0.152
Head^AAPM_HeadR_Spi_	2	127	5	1	139	.	0.221
Head^Brain_SAFIRE (Child)	5	151	15	20	150	7	0.865
Head^HeadRoutin (Child)	207	200	9	261	251	10	< 0.001
Head^InnerEar (Child)	40	107	2	10	103	5	0.671
Head^KAUH_Head_Fast_	2	136	5	6	142	3	0.402
Head^NeonateHead (Child)	5	157	18	6	172	11	0.584
NECK SOFT C-/+	13	160	12	15	183	28	0.908
Neck^KAUH_Neck_SAFIR	2	155	34	1	199	.	0.221
Neck^NeckRoutine (Child)	17	98	5	8	180	64	0.024
Thorax^1_Chest_Routine (Ch)	3	76	8	1	112	.	0.157
Thorax^Flash_Thorax_SAFIR	2	160	4	5	127	32	0.696

## Discussion

As a result of the COVID-19 pandemic, countless healthcare systems around the world were severely affected. Conversly, this event provided an interesting basis for valuable statistics. This retrospective study reviewed the radiation-dose distribution and routine scanning protocols for pediatric head computed tomography (CT) examinations at the Radiology Department of the King Abdulaziz University Hospital in Jeddah, comparing the data from a period at the height of the COVID-19 pandemic and a corresponding control period the year before.

To limit patient risks and exposure to the virus, clear instructions were issued to employees in the Radiology Department, including wearing masks and being tested before entering the department. Patients whose COVID-19 status was positive or suspected did not undergo imaging procedures. Instead, they were sent to the emergency room in accordance with infection control procedures [16]. A significant reduction in the number of endoscopic and surgical procedures has been observed as a result of the COVID-19 outbreak [17]. The majority of elective procedures were postponed or canceled in order to maintain the focus on COVID-19 patients and urgent surgeries. As a result of these measures, fewer elective diagnostic scans are performed. Currently, no studies were found discussing digital radiography rejection rates during the COVID-19 period in Saudi Arabia.

The total number of head CT scans decreased by approximately 5.6% during the pandemic, from 531 to 474. This reduction in number can be attributed to the shift in practice to cover only urgent requests. All other routine and appointment scans were canceled or delayed in order to shift the focus to COVID-19 cases. We noted that certain pediatric head imaging protocols were never performed during the COVID-19 period, such as cerebral angiograms, CT brain perfusion, CT venography, and brain navigation protocols. The absence of the latter can be explained by the lack of need for preoperative assessments due to the reduction of surgeries during the pandemic, whereas the shortage of requests for other protocols can be explained by probably decreased hospitalization rates. Staff shortage during the COVID-19 period also impacted the overall number of clinical examinations and imaging protocols. For instance, the CT technologists in our institution were four on a given day during COVID-19, three covering the day shifts and one the night shift. In contrast, during the control period, eight technologists were working on a given day, seven covering the day shifts and one during the nighttime.

The total mean DLP, CTDI_vol_, and exposure time decreased during the pandemic. This change can arguably be attributed to the desire to limit the contact between the technologist and the patient. In contrast, the scanning length increased during the COVID-19 period. The technologists being physically distant from their patients possibly accounted for this slight increase. Considering different regions in the world, pediatric head CT radiation doses before COVID-19 typically showed some variability; a study conducted in Turkey in 2019 involving 194 patients revealed that the median DLP values for head CT scans were 144.3, 233.7, 246.4, 288.9, for patients in the age groups of < 1 year, 1−5, 5−10, and 10−15 years, respectively [18]. Kanal et al. reported that the mean CTDIvol value was 27.3 mGy, and the mean DLP value was 390.9 mGy*cm [19], based on surveys distributed among 253 different hospitals in the USA in 2015.

It is worth mentioning that the presented study has limitations; this study was conducted in a single health center, thus further studies are required to support our findings with a multi-center study design. The current setting to conduct the study didn't take into consideration the age of patients scanned, and the variation of weight of patients scanned. 

## Conclusions

The scanning length, exposure time, and effective mAs showed significant reduction during the pandemic period in our institution; however, no significant change in radiation doses was noted for head and neck CT scans in pediatric patients during the COVID-19 pandemic. The significant reductions in these parameters may be attributed to the goal of reducing staff-patient contact time during the pandemic. We recommend further studies to evaluate the radiation doses and safety protocols after the peak of COVID-19, extending to a more current date. 
